# Study of messenger RNA inactivation and protein degradation in an *Escherichia coli *cell-free expression system

**DOI:** 10.1186/1754-1611-4-9

**Published:** 2010-07-01

**Authors:** Jonghyeon Shin, Vincent Noireaux

**Affiliations:** 1 Physics Department, University of Minnesota, 116 Church Street S.E., Minneapolis, MN 55455, USA

## Abstract

**Background:**

A large amount of recombinant proteins can be synthesized in a few hours with *Escherichia coli *cell-free expression systems based on bacteriophage transcription. These cytoplasmic extracts are used in many applications that require large-scale protein production such as proteomics and high throughput techniques. In recent years, cell-free systems have also been used to engineer complex informational processes. These works, however, have been limited by the current available cell-free systems, which are not well adapted to these types of studies. In particular, no method has been proposed to increase the mRNA inactivation rate and the protein degradation rate in cell-free reactions. The construction of *in vitro *informational processes with interesting dynamics requires a balance between mRNA and protein synthesis (the source), and mRNA inactivation and protein degradation (the sink).

**Results:**

Two quantitative studies are presented to characterize and to increase the global mRNA inactivation rate, and to accelerate the degradation of the synthesized proteins in an *E. coli *cell-free expression system driven by the endogenous RNA polymerase and sigma factor 70. The *E. coli *mRNA interferase MazF was used to increase and to adjust the mRNA inactivation rate of the *Firefly luciferase *(Luc) and of the enhanced green fluorescent protein (eGFP). Peptide tags specific to the endogenous *E. coli *AAA + proteases were used to induce and to adjust the protein degradation rate of eGFP. Messenger RNA inactivation rate, protein degradation rate, maturation time of Luc and eGFP were measured.

**Conclusions:**

The global mRNA turnover and the protein degradation rate can be accelerated and tuned in a biologically relevant range in a cell-free reaction with quantitative procedures easy to implement. These features broaden the capabilities of cell-free systems with a better control of gene expression. This cell-free extract could find some applications in new research areas such as *in vitro *synthetic biology and systems biology where engineering informational processes requires a quantitative control of mRNA inactivation and protein degradation.

## Background

Cell-free expression has become a serious alternative to cell-based expression. In response to an increasing number of applications that require fast production of a large amount of proteins [1], new preparation methods and new reaction components are frequently proposed to improve protein productivity of cell-free systems and to reduce the cost of reaction [2,3]. These systems use bacteriophage transcriptions, such as T7, and extracts with low degradation of both mRNAs and proteins to produce on the order of 1 mg/ml of proteins in batch mode after a few hours of incubation. As they become more powerful, cell-free systems are used in new applications. *In vitro *synthetic biology is one of the new research areas where transcription-translation extracts can be employed to engineer processes based on biological information. Cell-free elementary gene circuits [4,5], pattern formation [6] and synthetic vesicles [7,8] have been engineered with cell-free systems. However, the properties of conventional cell-free expression systems are not well adapted for this type of study that requires more than a fast and a powerful expression of proteins. In particular, the control of mRNA inactivation and protein degradation rates are essential components for the construction of interesting informational processes *in vitro*. The production of cycles in time or patterns in space requires a precise balance between a source and a sink [5,9]. It is a general and an essential property of dynamical systems. Whereas most of the efforts to optimize cell-free systems have consisted in increasing protein productivity, no procedure has been proposed to change the inactivation rate of mRNAs and the degradation rate of proteins in cell-free expression systems. It is one of the main bottlenecks for the development and the study of quantitative informational processes *in vitro*.

In this work, an approach is presented to accelerate the global mRNA turnover of the synthesized mRNAs in a cell-free reaction and to control the degradation of the synthesized proteins. The *E. coli *mRNA interferase MazF was used to adjust the inactivation rate of synthesized mRNAs and the endogenous *E. coli *AAA + proteases were used to control the degradation of synthesized proteins in a cell-free expression system driven by the endogenous *E. coli *RNAP and sigma factor 70 [10]. The experiments were carried out with eGFP and deGFP, a highly translatable version of eGFP with the same fluorescence properties [10]. The *Firefly luciferase *was used as a second reporter protein for control experiments.

MazF-MazE is a toxin-antitoxin pair found in *E. coli*. The antitoxin MazE inhibits the activity of the toxin MazF. MazF is a small ribonuclease that inactivates mRNAs by cleaving at the ribonucleotide ACA single strand sequence [11]. The toxin is expressed in *E. coli *under special conditions, such as amino acid starvation, to block protein synthesis by inactivating all the mRNAs. Ribosomes and tRNAs are not inactivated by MazF. For these reasons, we found that MazF was a convenient tool to accelerate the global mRNA turnover in a cell-free reaction without inactivating other types of RNA. The mean lifetime of deGFP mRNA, modeled by an exponential decay, could be easily tuned from 13 minutes (the mean lifetime without addition of toxin), to 0 minutes (complete inactivation). Practically, the mRNA mean lifetime was adjusted by adding a small fraction of an extract containing MazF to a cell-free reaction.

A complete control of gene expression dynamics *in vitro *also requires adjusting the degradation rate of the synthesized proteins while not affecting transcription and translation machineries. This aspect is of particular importance to prevent synthesized proteins from accumulating in batch mode reaction. The endogenous AAA + proteases, such as the ClpXP and ClpAP complexes, present in the *E. coli *extract provide an adequate solution. Synthesized proteins have to be tagged either in N-terminal or C-terminal with a short amino acid sequence to be specifically degraded by the ClpXP or ClpAP complexes [12]. In this study, seven tags were tested on deGFP. With the 11-residue SsrA tag [13], the deGFP degradation rate in cell-free reaction was constant up to a concentration of one micromolar. We show that cell-free production of deGFP could be predicted when MazF and the SsrA tag were used concurrently.

## Methods

### Extract preparation

The cell-free system used in this study has been developed by Shin and Noireaux [10]. Briefly, the crude extract was prepared from *E. coli *BL21 Rosetta2 cells according to Kigawa et al [14] and Liu et al [15] with slight modifications. S30 buffer A (50 mM Tris, 60 mM potassium glutamate and 14 mM magnesium glutamate, pH 7.7, 2 mM DTT) was used for washing and resuspension. The crude extract was dialyzed against S30 buffer B (5 mM Tris, 60 mM potassium glutamate and 14 mM magnesium glutamate, pH 8.2, 1 mM DTT). The cells were broken with a bead beater (mini bead-beater-1, Biospecs Products Inc, Bartlesville, OK). The crude extract was stored at -80 C after dialysis. The endogenous *E. coli *RNA polymerase was used for expression. Preparation of the MazF crude extract was identical to the preparation of the crude extract for cell-free reaction except for the expression of MazF prior to preparation. The MazF gene was obtained by PCR using *E. coli *as a template and cloned under the arabinose promoter in the plasmid pBAD/His A (Invitrogen). At OD600 = 1.2, cells bearing the MazF plasmid were induced with 0.25% arabinose (final concentration) for one hour before preparing the extract. A typical concentration of 27-30 mg/ml and 22 mg/ml of proteins in the crude extract, with and without MazF respectively, was measured by Bradford assay. Both crude extracts are stable at least 1 year when stored at -80°C.

### Cell-free reaction

The standard cell-free reactions were composed of 33% crude extract (between 9 and 9.5 mg/ml of proteins), the other 66% containing the reaction buffer and plasmid with the following final concentrations: 50 mM Hepes pH 8, 1.5 mM ATP and GTP each, 0.9 mM CTP and UTP each, 1 mM spermidine, 0.75 mM cAMP, 0.33 mM NAD, 0.26 mM coenzymeA, 30 mM 3-phosphoglyceric acid, 0.068 mM folinic acid, 0.2 mg/ml tRNA, 1 mM IPTG, 1.5 mM amino acids. The concentrations of PEG 8000, magnesium glutamate and potassium glutamate were adjusted depending on the reporter used [10]. The cell-free reactions with the MazF crude extract were composed of 43% MazF crude extract (between 9 and 9.5 mg/ml of proteins), the other 57% containing the reaction buffer and plasmid with the same final concentrations as the standard cell-free reactions. The plasmid concentration was adjusted depending on the experiment (a final concentration comprised between 0.1 nM and 5 nM was used in this study). The reactions were incubated at 22°C for Luc and 29°C for eGFP. The reagents used for cell-free reactions were purchased from Sigma, USB Corporation (GTP, CTP, UTP) and Roche (tRNA, amino acids). Other reagents used in this study: Ribonuclease A (Sigma), Tagetin (Epicentre Biotechnologies), MazF (Takara Bio Inc).

### Protein expression and purification

The plasmid pET21a(+) (Novagen) was used for recombinant protein expression. The proteins His-MazE (6Histag in N-terminal), His-eGFP-SsrA (6Histag in N-terminal and SsrA tag in C-terminal) and His-eGFP-SsrA-DD (6Histag in N-terminal and SsrA-DD tag in C-terminal) were over-expressed in *E. coli *BL21 (DE3) and purified by affinity chromatography on agarose nickel beads according to the manufacturer protocol (Invitrogen). The proteins were desalted against a storage buffer (50 mM Tris HCl pH 7.5, 5% glycerol) and stored at -80°C. The concentration of the purified proteins was measured by Bradford assay. Pure recombinant eGFP (Clontech) was used to determine the concentration of His-eGFP-SsrA and His-eGFP-SsrA-DD.

### Plasmid preparation

All the plasmids used in this study originate from the plasmid pBEST-Luc (Promega). The list and sequences of the different regulatory parts are reported in the additional file 1. Luc refers to *Firefly luciferase *[GenBank: CAA59281.1], eGFP to the enhanced green fluorescent protein [GenBank: CAD97424.1], deGFP to eGFP-Del6-229 (enhanced green fluorescent protein truncated and modified in N- and C-terminal, [10]), UTR1 to the untranslated region containing the T7 g10 leader sequence for highly efficient translation initiation [16] [GenBank: M35614.1], T500 to the transcription terminator [17], OR2-OR1-Pr to the lambda repressor Cro promoter [GenBank: J02459.1], SsrA, SsrA-D, SsrA-DD, Crl, YbaQ, YdaM and OmpA to the tags specific to the ClpXP and ClpAP complexes [12]. The plasmids were prepared using the standard molecular cloning procedures. Picogreen (Invitrogen) was used to measure plasmid concentration.

### Measurements

The fluorescence measurements (kinetics and end-point) were either performed with a Wallac Victor III plate reader (PerkinElmer, 384-well plate) or with an Olympus IX-71 inverted microscope equipped with a photo multiplier tube (Hamamatsu, H7421-40). Pure recombinant eGFP (Clontech) was used for calibration and quantification. Luc expression was measured with a custom-built luminometer [4]. Pure Luc and Luc assay kit (Promega) were used for calibration and measurements.

## Results and discussion

### Luc and eGFP maturation time

**Figure 1:**
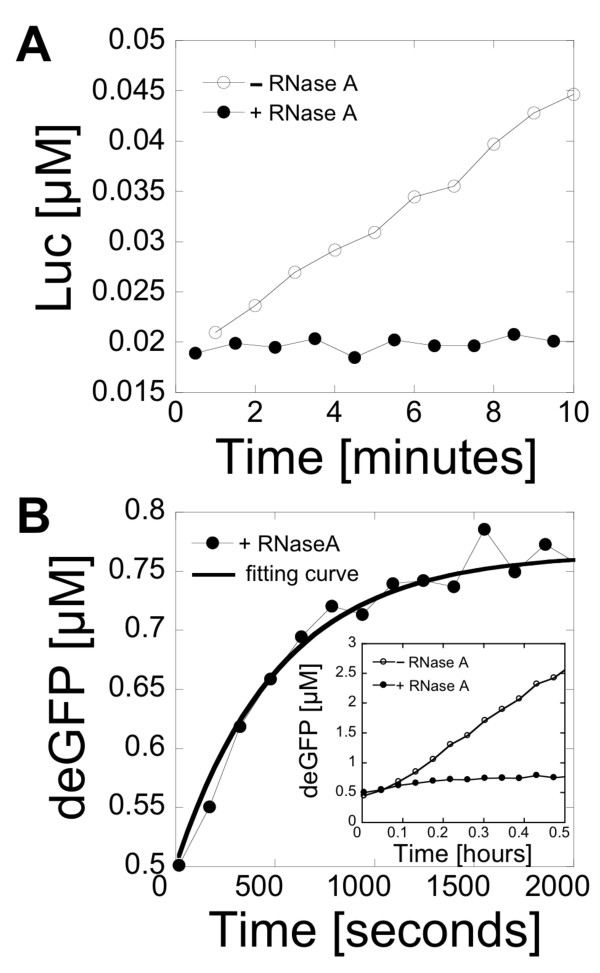
**Maturation time of Luc and deGFP in a cell-free system**. RNase A was added to cell-free reactions after 30 minutes of incubation to stop transcription and translation. (A) Kinetics of Luc expression (1 nM plasmid pBEST-UTR1-Luc) with and without addition of RNase A. (B) Kinetics of deGFP expression (5 nM plasmid pBEST-OR2-OR1-Pr-UTR1-deGFP-T500) after addition of RNase A. Inset: kinetics of deGFP expression (5 nM plasmid pBEST-OR2-OR1-Pr-UTR1-deGFP-T500) with and without addition of RNase A on a larger time scale.

The experiments were carried out with a cell-free expression system optimized for high protein production [10]. This cell-free system uses the endogenous *E. coli *RNA polymerase and sigma factor 70 for transcription. The reporter proteins Luc, eGFP and deGFP were used in this study. deGFP, described previously as eGFP-Del6-229 [10], is a highly translatable version of the original eGFP under *E. coli *promoters with the same fluorescence properties as eGFP. These reporters allow precise quantification of gene expression in cell-free systems. Before characterizing mRNA inactivation and protein degradation in a cell-free reaction, the maturation time of Luc and deGFP was determined with an assay based on the ribonuclease A (RNase A). RNase A is a powerful endonuclease that degrades single strand RNA. The enzyme was added to cell-free reactions to stop gene expression in a minimum amount of time by degrading mRNA and ribosomes. The maturation times of Luc and deGFP were determined from the increase of luminescence and fluorescence, respectively, after complete inhibition of gene expression. Addition of a small amount of RNase A was necessary to inhibit gene expression in cell-free reactions (see Figure S1, additional file 1). In the first experiment, RNase A was added at a final concentration of 600 nM to a reaction containing the plasmid pBEST-UTR1-Luc after 30 minutes of incubation at room temperature. No increase of luminescence was observed after addition of the ribonuclease (Figure 1A). In the control reaction, with no RNase A, a fast increase of luminescence was measured. The maturation time of synthesized Luc in our cell-free system was less than a minute. The experiment with Luc validated the RNase A assay as a method to stop gene expression almost instantly. The same experiment was done with deGFP. RNase A was added to a cell-free reaction containing the plasmid pBEST-OR2-OR1-Pr-UTR1-deGFP-T500 after 30 minutes of incubation at 29°C. The increase of fluorescence measured after addition of the ribonuclease was well fitted by a first order reaction (Figure 1B, equation (1.1)):(1.1)(1.2)

where deGFP_f _is the fluorescent deGFP, deGFP_d _is the dark deGFP (the primary amino acid chain is synthesized but the reporter protein is not fluorescent), [deGFP_0_] = [deGFP_f_] + [deGFP_d_] = constant and 1/κ is the maturation time that includes folding of the protein and formation of the fluorophore by oxidation [18]. Equation (1.2) is the solution to equation (1.1). Fitting of the data gave a reproducible maturation time of 1/κ = 8-8.5 minutes for deGFP in our cell-free extract. This maturation time was taken into account in all of the subsequent calculations. The same maturation time was obtained when the experiment was carried out after 1 hour of incubation at 29°C. This result was comparable to the maturation time of eGFP measured *in vivo *[19].

### Endogenous messenger RNA inactivation

**Figure 2:**
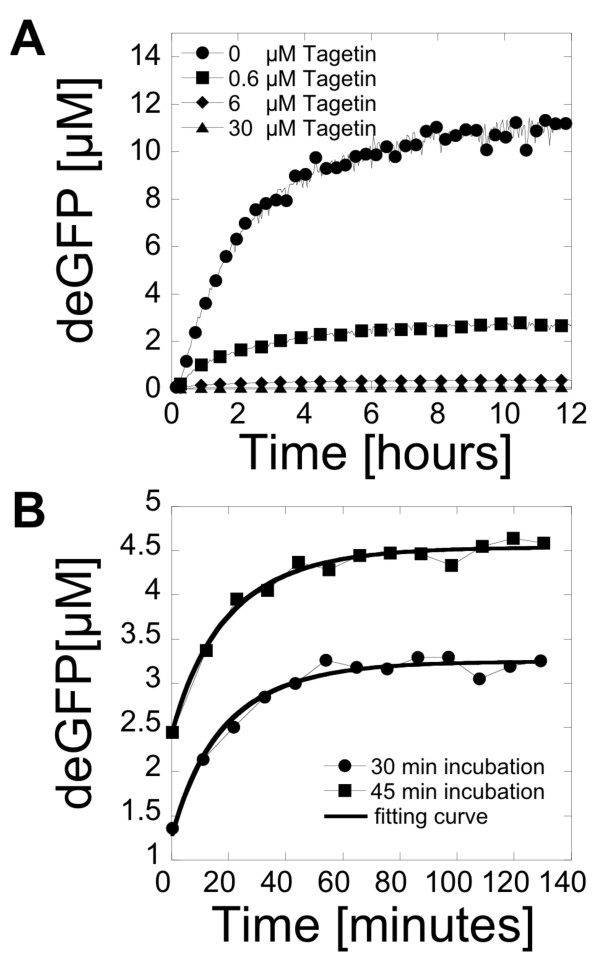
**deGFP messenger RNA lifetime in a cell-free reaction**. The endogenous mRNA inactivation rate was measured with the reporter deGFP in an assay based on Tagetin, a RNA polymerase inhibitor. (A) Kinetics of deGFP expression (5 nM pBEST-OR2-OR1-Pr-UTR1-deGFP-T500) as a function of Tagetin concentration. Tagetin was added at the beginning of reaction. (B) Kinetics of deGFP expression (5 nM pBEST-OR2-OR1-Pr-UTR1-deGFP-T500) after addition of Tagetin (30 μM). Tagetin was added to the cell-free reactions after 30 and 45 minutes of incubation.

Degradation is an essential feature of gene expression dynamics. In *E. coli*, the global mRNA half-life has been estimated to be 6.8 minutes [20]. *E. coli *cell-free extracts contain almost all of the cytoplasmic components of the cells including the proteins responsible for degradation of mRNAs. Many enzymes contribute to mRNA decay *in vivo *which makes mRNA degradation a complex process [21]. Furthermore, mRNA degradation is different for each gene since it depends on the sequence and the structure of transcripts. It is important to make a distinction between inactivation, which is a loss of function, and degradation, which is a loss of mass. In this work, inactivation of mRNA was measured rather than degradation. Therefore, decay and lifetime refer to inactivation. The rate of mRNA inactivation in a cell-free reaction is expected to be smaller than *in vivo *since the extract is diluted ten to twenty times compared to real cytoplasms. A complete study of mRNA inactivation would require characterizing the inactivation of each gene used in cell-free reactions with specific techniques such as Northern blot hybridization. Instead, we estimated the deGFP mRNA inactivation rate in the cell-free reaction by modeling the inactivation as an exponential decay. This model, already used to measure the global mRNA half-life in *E. coli *[20], bypasses the biochemical details of the inactivation process. A simple procedure was used that consists in blocking transcription in a running cell-free reaction. The mRNA inactivation rate was determined from the accumulation of the reporter protein in the solution after transcription was stopped. This assay captures the average mRNA inactivation time independently of the detailed enzymatic mechanisms. The RNA polymerase inhibitor Tagetin was used to stop transcription [22]. First, the amount of Tagetin necessary to stop transcription completely with no leak was determined by adding the inhibitor at different concentrations right at the beginning of expression (Figure 2A). The fluorescence signal stayed at the background level with a final Tagetin concentration of 30 μM. This concentration of inhibitor was used in cell-free reactions after 30 and 45 minutes of incubation and deGFP fluorescence was recorded (Figure 2B). This assay was modeled by the following set of equations:(1.3)(1.4)(1.5)(1.6)

where deGFP_f _is the fluorescent deGFP, deGFP_d _is the dark deGFP, m is the concentration of deGFP mRNA, m_0 _is the concentration of active mRNAs when transcription is stopped, 1/κ is the maturation time that includes folding of the protein and formation of the fluorophore by oxidation, β is the mRNA inactivation rate and α is the protein production rate. This model describes mRNA inactivation as an exponential decay [20]. The reference time point t = 0 is set to transcription arrest (addition of Tagetin). The initial concentration of deGFP_f _is set to zero. The initial concentration of deGFP_d _is set to 0.25 μM (this concentration is given by Figure 1B). Equation (1.6) is the solution to the equations (1.3), (1.4) and (1.5). The data were fitted to equation (1.6). No estimations of α and m_0 _have been determined in this work. The determination of β was independent of the product αm_0_. No interesting information could be extracted from the numerical constant αm_0 _since it depends on the time at which the assay is performed (due to m_0_). With a maturation time of 8 minutes for deGFP, we found an endogenous deGFP mRNA inactivation rate β of 0.077 min^-1^, which corresponds to an average lifetime of 13 minutes. This result is comparable to some estimation of mRNA lifetime in a commercial cell-free system [23].

### Messenger RNA inactivation with MazF

**Figure 3:**
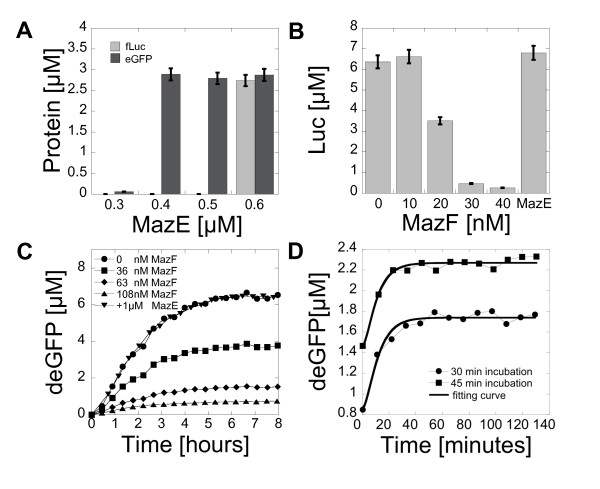
**Increase of the messenger RNA inactivation rate with the MazF interferase in a cell-free system**. (A) Addition of pure MazE into a MazF cell-free reaction (MazF extract). End-point measurements of Luc production (5 nM pBEST-UTR1-Luc) and deGFP production (5 nM pBEST-UTR1-eGFP). (B) End-point measurements of Luc synthesized in a standard cell-free reaction (no MazF) as the function of MazF added to the reaction (5 nM pBEST-UTR1-Luc). The last bar is a control experiment with 140 nM MazF and 1 μM MazE. (C) Kinetics of deGFP expression (1 nM pBEST-OR2-OR1-Pr-UTR1-deGFP-T500) with a range of MazF. The MazE sample was tested with 90 nM MazF. (D) Kinetics of deGFP expression (5 nM plasmid pBEST-OR2-OR1-Pr-UTR1-deGFP-T500) with 36 nM of MazF after addition of 30 μM Tagetin.

An important bottleneck for the construction of complex informational processes *in vitro *with cell-free expression systems is the lack of simple procedures to change the global mRNA inactivation turnover. Whereas the synthesis rate of mRNA can be easily tuned by changing the promoter strength, no simple method has been proposed to increase the inactivation rate of transcripts. Acceleration of the mRNA turnover is of particular interest since mRNA inactivation in cell-free reactions is slow. We used the *E. coli *interferase MazF to increase and to adjust the mRNA inactivation rate in cell-free reactions. MazF inactivates transcripts exclusively, without inactivating or degrading other types of RNAs such as ribosomal and transfer RNAs [11]. MazF inhibits protein expression by cleaving mRNAs bearing any ACA single strand ribonucleotide sequence. When MazF is expressed in *E. coli*, no protein is synthesized [11]. To make the method cost-effective and easy to use, a cell-free extract containing the toxin was prepared. The toxin was expressed in the cells before preparing the extract. As expected, *E. coli *cell growth was stopped after induction of MazF expression (see Figure S2, additional file 1). Preparation of the MazF extract was identical to the preparation of the extract used for cell-free expression [10]. In cell-free reactions using only the MazF crude extract (composed of 43% crude extract and 57% of buffer-nutrients, see Methods), full expression of Luc and deGFP was recovered with a concentration of 0.6 μM and 0.4 μM MazE respectively (Figure 3A). The concentration of MazF in the crude extract was estimated from the concentration of antitoxin MazE required to recover the complete expression using a stoichiometry MazF:MazE 2:1 [24]. Based on these numbers, the concentration of MazF in the MazF crude extract was between 1.8 μM (eGFP) and 2.8 μM (Luc). These values of MazF concentrations were used in the following experiments. To increase and to adjust the mRNA inactivation rate in a cell-free reaction, two approaches were possible: addition of pure MazE in the MazF crude extract as shown in Figure 3A or addition of MazF extract into a standard cell-free reaction that does not contain MazF. The experiments were performed using the second approach for two reasons: it allowed a much better control of the mRNA decay rate and the protein production in standard cell-free reactions (extract with no MazF) was twice as much. The messenger RNA inactivation rate could be fully tuned by the addition of a small fraction of MazF crude extract for both Luc (Figure 3B) and deGFP (Figure 3C). Luc and deGFP productions were reduced by half with a final MazF concentration of 56 nM and 36 nM, respectively. The protein production was entirely recovered when MazE was added into the reaction. This control experiment confirmed that the increase of the mRNA inactivation rate was specific to MazF. The same Tagetin assay and the same model (equations (1.3), (1.4), (1.5), (1.6)) were used to determine the mRNA lifetime when MazF was present in the reaction. Figure 3D shows an example of an mRNA decay measurement. In this experiment, a concentration of 36 nM MazF was used in a cell-free reaction containing the plasmid pBEST-OR2-OR1-Pr-UTR1-deGFP-T500. The total deGFP production was reduced by a factor of two with an mRNA lifetime of 5 minutes. With this method, the deGFP mRNA lifetime could be set precisely from endogenous level (13 minutes) to complete inactivation (0 minute). It is important to mention that MazF is commercially available. However, no effect of the commercial enzyme was observed in our cell-free reactions. The commercial MazF was not functional for unknown reasons (see Figure S3, additional file 1). Preparation of a crude MazF extract based on the T7 bacteriophage transcription was also tested. Titration with the antitoxin MazE did not show any major difference compared to the results obtained with the cell-free system used in this study based on the endogenous transcription machinery (see Figure S4, additional file 1).

### Protein degradation

**Figure 4:**
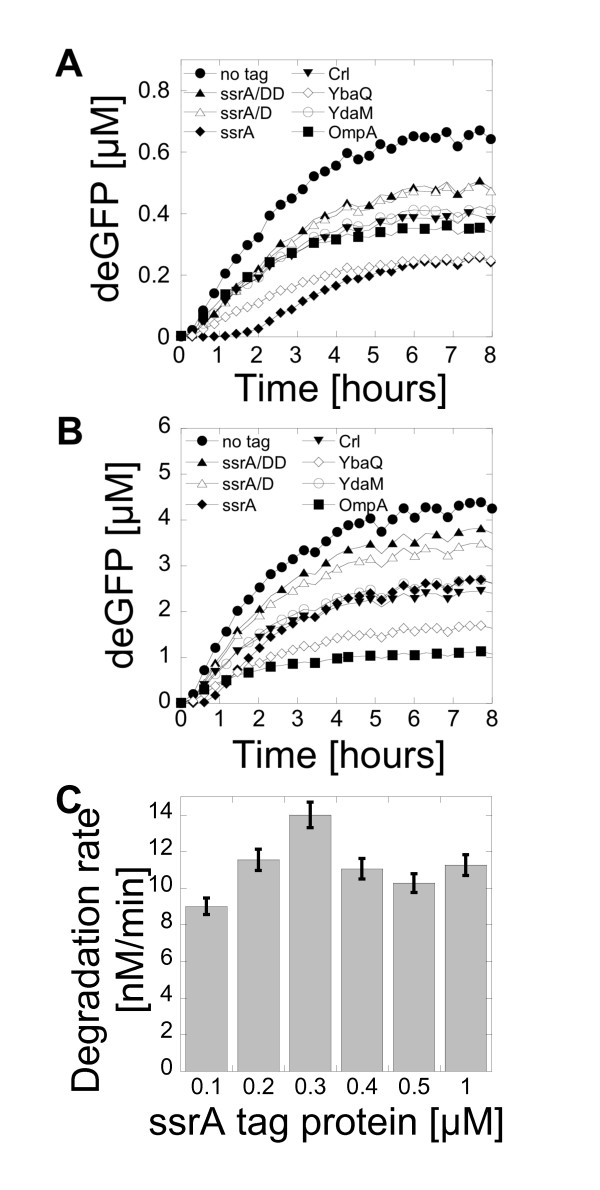
**Protein degradation with the AAA + proteases in a cell-free system**. (A) Kinetics of deGFP expression with 0.1 nM plasmid pBEST-OR2-OR1-Pr-UTR1-deGFP-Tag-T500. (B) Kinetics of deGFP expression with 1 nM plasmid pBEST-OR2-OR1-Pr-UTR1-deGFP-Tag-T500. (C) Degradation rate of pure His-eGFP-SsrA protein at different concentrations in cell-free reactions.

Cells have to regulate their internal environment to maintain stable and constant conditions, a property named homeostasis. Degradation of proteins is not only essential for homeostasis, it is also necessary for gene regulation processes. In bacteria, accumulation of proteins in the cytoplasm is prevented by a general proteolysis and a regulatory proteolysis that eliminate misfolded, denaturated or incomplete polypeptides [25]. In living organisms, proteins are also cleared by division and volume expansion, which is a significant advantage compared to a batch mode cell-free reaction with a constant volume. In *E. coli*, specific degradation of cytoplasmic proteins is achieved by AAA + proteases such as the ClpXP and ClpAP complexes. Degradation tags, also named degrons, are either part of or added to the proteins to direct their degradation [12]. Modern *E. coli *cell-free expression systems are prepared from strains with low proteolysis activity. The extract used in this study was prepared from the strain BL21 Rosetta2, which is deficient in the two main proteases Lon and OmpT. No degradation was observed when pure eGFP and pure Luc were added to the cell-free reaction (see Figure S5, additional file 1). We used the endogenous AAA + proteases present in the extract to carry out specific degradation of the synthesized proteins in our cell-free reactions. The AAA + proteases activity was characterized with deGFP. Sequences of seven degrons recognized by AAA + proteases were fused to the reporter gene by cloning: OmpA (N-terminal) and six C-terminal tags, SsrA, SsrA-DD (SsrA tag with two mutations that prevent degradation), SsrA-D (SsrA tag with one mutation), Crl, YbaQ and YdaM [12] (list of sequences is reported in the additional file 1). Expression of the tagged deGFP was compared to deGFP at two different plasmid concentrations, 0.1 nM (Figure 4A) and 1 nM (Figure 4B), in a cell-free reaction with no MazF. At a plasmid concentration of 0.1 nM, the SsrA tag was the most efficient with approximately 60% of the proteins degraded by the end of the reaction. At 1 nM plasmid, the OmpA tag was the most efficient with 75% of the proteins degraded by the end of the reaction. Protein degradation efficiency with the seven degrons was the same at 0.1 nM and 1 nM plasmid concentration except for the SsrA and the OmpA degrons. The seven tags tested in this work allowed setting protein degradation to different rates as shown in the Figure 4A and 4B. However, different degrons could be used to adjust the degradation rate [12]. To determine the protein degradation rate, eGFP was purified with the SsrA tag (His-eGFP-SsrA) and with the non-degradable tag SsrA-DD (His-eGFP-SsrA-DD) in C-terminal. The two reporter proteins were added to separate cell-free reactions containing no plasmid and the fluorescence was recorded over time (see Figures S5 and S6, additional file 1). Without a degradation tag, eGFP was not degraded whereas a net degradation was observed for His-eGFP-SsrA. The degradation rate, obtained by measuring the slope of the degradation curves, was constant up to a concentration of 1 μM with an average of 10 nM.min^-1 ^(Figure 4C). At a concentration of 10 μM proteins, the degradation rate was 4 nM.min^-1 ^(data not shown). This result could serve as a reference to estimate the degradation rate related to the other degrons by using Figures 4A and 4B. Protein degradation with the AAA + proteolytic pathway was also tested in a T7 transcription based cell-free system. No major differences were observed compared to the results obtained with the extract used in this study based on the *E. coli *endogenous transcription machinery (see Figure S7, additional file 1).

### Concurrent messenger RNA inactivation and protein degradation

**Figure 5:**
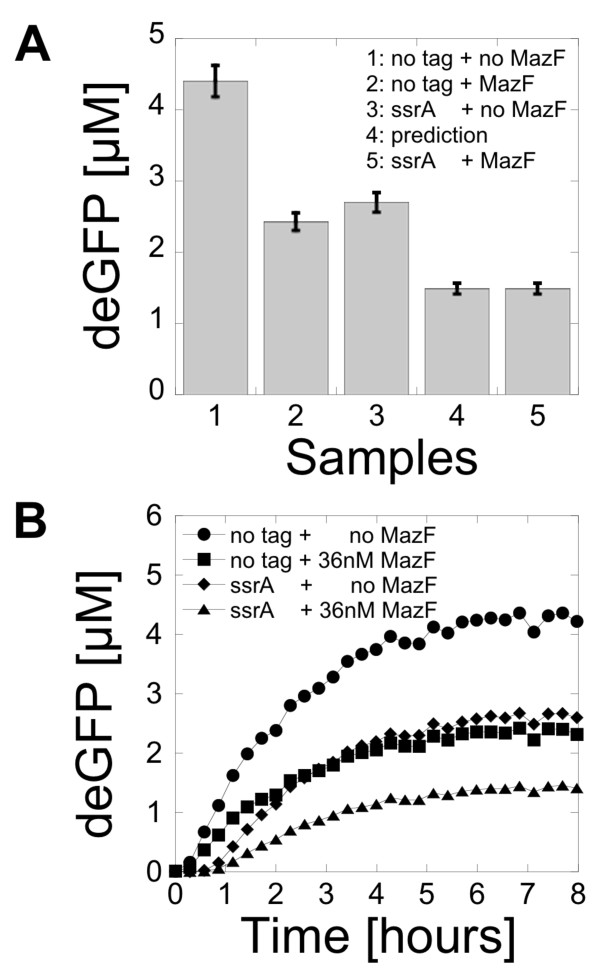
**Concurrent mRNA inactivation and protein degradation in a cell-free system**. (A) End-point measurement of deGFP expression (1 nM pBEST-OR2-OR1-Pr-UTR1-deGFP-T500 or 1 nM pBEST-OR2-OR1-Pr-UTR1-deGFP-SsrA-T500). 1) no degradation (0 nM MazF, deGFP), 2) mRNA inactivation (36 nM MazF, deGFP), 3) protein degradation (0 nM MazF, deGFP-SsrA), 4) expected protein production with mRNA inactivation (36 nM MazF) and protein degradation (deGFP-SsrA), 5) protein production measured with mRNA inactivation (36 nM MazF) and protein degradation (deGFP-SsrA). (B) Kinetics of expression for the different cases (1 nM pBEST-OR2-OR1-Pr-UTR1-deGFP-T500 or 1 nM pBEST-OR2-OR1-Pr-UTR1-deGFP-SsrA-T500).

Increase of the mRNA inactivation rate with MazF and acceleration of the protein degradation with AAA + proteases were carried out concurrently in the same reaction. The goal was to determine if the protein production could be predicted from the characterization of each process measured separately. Expression of deGFP was carried out with no MazF as a control experiment. First, two separate reactions were carried out, one with an increase of the deGFP mRNA inactivation rate (36 nM MazF, deGFP) the other one with protein degradation (deGFP-SsrA, 0 nM MazF, no increase of the mRNA inactivation rate). The protein production ratio, obtained by comparing the protein production at the end of each separate reaction to the protein production with no inactivation and no degradation mechanism, was used to predict the protein production of deGFP-SsrA in a cell-free reaction also including an increase of the mRNA inactivation rate (36 nM MazF). The predicted protein production, obtained by multiplying the protein production ratios of each separate experiment, was compared to the experimental production of deGFP-SsrA with 36 nM MazF. Figure 5A shows the end-point measurement and Figure 5B shows the kinetics. The prediction matched the result of the experiment within 1% of the total protein production. This experiment showed that the effects of the increase of the mRNA inactivation rate with MazF and the acceleration of protein degradation with AAA + degrons add linearly when they are carried out simultaneously.

## Conclusions

In this work, the inactivation rate of mRNA and the degradation rate of proteins have been studied in a transcription-translation cell-free reaction. Methods to increase the inactivation rate of synthesized mRNAs and to induce the degradation rate of synthesized proteins have been described. These methods are quantitative, cost-effective and simple to use. Inactivation and degradation were characterized with a cell-free expression system driven by the endogenous *E. coli *RNA polymerase, which presents some advantages for the construction of synthetic circuitry *in vitro*. Structure of *E. coli *promoters provides much more modularity to engineer informational processes, such as gene circuits, compared to bacteriophage promoters used in conventional cell-free systems. Construction of any interesting synthetic circuitry, such as cycles in time or patterns in space, requires a fine balance between the source and the sink [5,9]. This work is a first step to provide the necessary inactivation and degradation tools to engineer complex informational processes *in vitro *involving transcription and translation reactions. For instance, it would be interesting to test the production of oscillations *in vitro *with this system. On a broader perspective, the introduction of quantitative sinks contributes to the development of cell-free toolboxes to synthesize, to run and to study informational processes outside living organisms.

## Abbreviations

(Luc): *Firefly luciferase*; (eGFP): enhanced green fluorescent protein; (RNase A): Ribonuclease A; (eGFP-Del6-229, see [10]): deGFP.

## Competing interests

The authors declare that they have no competing interests.

## Authors' contributions

JS carried out all the experiments. VN carried out the background work of extract preparation and optimization. VN prepared the draft of the manuscript. All authors read and approved the final manuscript.

## Supplementary Material

Additional file 1 **supplementary information for Shin and Noireaux "Study of mRNA inactivation and protein degradation in an *Escherichia coli *cell-free expression system"**. supplementary information includes a list of the sequences, data on RNase A and on MazF, control experiments on protein degradation, control experiments with a T7 transcription-based cell-free system. Click here for file
