# Supplementary material for: Study of messenger RNA inactivation and protein degradation in an Escherichia coli cell-free expression system
Source: J Biol Eng. 2010 Jul 1;4:9. doi: 10.1186/1754-1611-4-9 (PMC2907309; doi:10.1186/1754-1611-4-9)
Supplement: Additional file 1 — supplementary information for Shin and Noireaux "Study of mRNA inactivation and protein degradation in an Escherichia coli cell-free expression system". supplementary information includes a list of the sequences, data on RNase A and on MazF, control experiments on protein degradation, control experiments with a T7 transcription-based cell-free system. [file 1754-1611-4-9-S1.PDF]

List of genes and regulatory parts used for plasmid constructions

Plasmid pBEST-Luc (Promega) was the original plasmid used in this work for cloning.

PtacI:

TTGACAATTAATCATCGGCTCGTATAATGTGTGGAATTGTGAGCGGATAACAATT

OR2-OR1-Pr:

TGAGCTAACACCGTGCGTGTTGACAATTTTACCTCTGGCGGTGATAATGGTTGCA

UTR1:

AATAATTTTGTTTAACTTTAAGAAGGAGATATA

Luc:

ATGGAAGACGCCAAAAACATAAAG……AAGGGCGGAAAGTCCAAATTGTAA

eGFP:

ATGGTGAGCAAGGGCGAGGAGCTGTTCACCGGGGTGGTGCCCATCCTGGTCGAGCT
GGACGGC…………GTCCTGCTGGAGTTCGTGACCGCCGCCGGGATCACTCTCGGCAT
GGACGAGCTGTACAAGTAA

eGFP-Del6-229 (re-named deGFP):

ATGGAGCTTTTCACTGGCGTTGTTCCCATCCTGGTCGAGCTGGACGGC……….GTCCT
GCTGGAGTTCGTGACCGCCGCCGGGATCTAA

T500:

CAAAGCCCGCCGAAAGGCGGGCTTTTCTGT

SsrA:

GCAGCAAACGACGAAAACTACGCTTTAGCTGCT

SsrA/D:

GCAGCAAACGACGAAAACTACGCTTTAGATGCT

SsrA/DD:

GCAGCAAACGACGAAAACTACGCTTTAGATGAC

Crl:

TTTCGTGATGAACCTGTTAAACTTACCGCC

YbaQ:

AGAAGGGAAGAAAGAGCAAAGAAGGTAGCA

YdaM:

TGCAAGAATGATGGAAGAAATAGGGTACTAGCAGCA

OmpA:

AAAAAAACTGCTGCTATCGCGATCGCGGTC

Expression as a function of Ribonuclease A

Ribonuclease A was purchased from Sigma. Expression stays at background level with any concentration of RNase A tested. We used a concentration of 600 nM in our experiments.

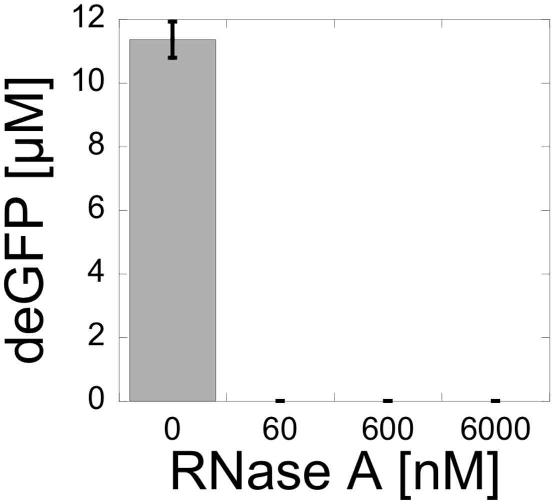

Figure S1
End-point measurement of deGFP synthesized as a function of Ribonuclease A. RNase A was added right at the beginning of the cell-free reactions (5nM plasmid pBEST-OR2-OR1-Pr-UTR1-deGFP-T500, 4 mM Mg-glutamate, 60 mM K-glutamate, 1.5 mM each amino acids, 2% PEG8000, 3-PGA buffer).

Effect of MazF on *E. coli* cells growth

The MazF gene was obtained by PCR from *E. coli* and cloned into the plasmid pBAD/His A (Invitrogen). The plasmid was named pBADmod1-linker1-MazF. The blank plasmid without MazF gene was named pBADmod1-linker1. The two plasmids were transformed into the *E. coli* strains BL21 AI (Invitrogen) and BL21 RIL (Novagen).

The cells were grown in LB at 37°C with ampicillin as the antibiotic. Expression of MazF was induced by the addition of 0.2% arabinose (final concentration) into the medium. As expected, expression of MazF inside the cells was followed by a growth arrest.

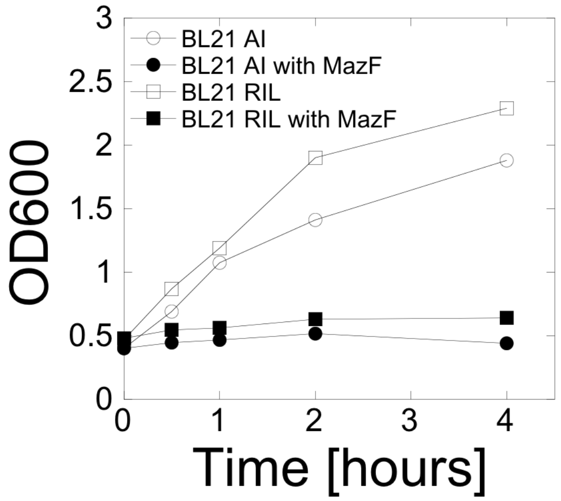

Figure S2
***E. coli* cell growth after induction with 0.2% arabinose to express MazF.**

Activity of commercial MazF

Pure MazF was purchased from Takara Bio Inc. The cell-free reactions were performed with T7 RNAP, plasmid pIVEX2.3d-eGFP and PEP buffer. The reaction was composed of:

- 50 µl extract
- 30 µl buffer 5X (PEP buffer)
- 5 µl potassium glutamate at 3 M (100 mM final)
- 6.7 µl magnesium glutamate at 180 mM (8 mM final)
- 17 µl amino acids at 3 mM (Roche mix, 0.33 mM final each)
- 3 µl pIVEX2.3d-eGFP at 50 nM (1 nM final)
- 23.3 µl water

The reaction was split into 7*18 µl samples and 2µl of commercial MazF were added (20 µl total volume for each reaction).

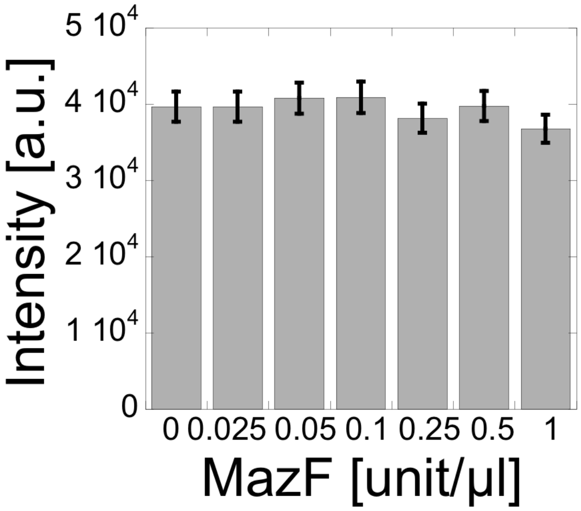

Figure S3
Expression as a function of commercial interferase MazF. End-point measurements of eGFP synthesized as a function of commercial interferase MazF added into the reaction. Fluorescent intensity was measured on plate reader. No effects were observed. Even with the highest concentration, protein production did not decrease.

mRNA degradation with MazF in a T7 cell-free system

A cell-free expression system with the T7 RNAP was used to test mRNA inactivation with MazF. This cell-free is the same as the one used in this work except that it contains the T7 RNA polymerase.

A range of pure MazE was used to recover expression of eGFP from the plasmid pIVEX2.3d-eGFP in a MazF extract. The plasmid concentration was fixed to 2 nM. The experiment was carried out at 30C, on plate reader.
As we can see in the Figure S4, the results obtained with a T7 system are comparable to results obtained with the extract used in the study (transcription with the *E. coli* RNA polymerase and sigma factor 70, see Figure 3A).

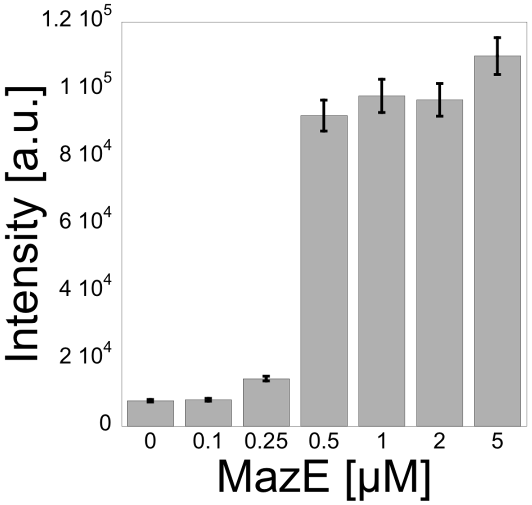

Figure S4
Cell-free expression as a function of pure MazE in a MazF extract with the T7 bacteriophage RNA polymerase for transcription. End-point measurements of eGFP synthesized as a function of MazE added to the reactions. Fluorescent intensity was measured on plate reader after 4 hours.

Degradation of pure Luc and pure eGFP in a cell-free reaction

Pure Luc (Promega) and pure His-eGFP-SsrA-DD were added to a cell-free reaction (one reaction for each). Luminescence and fluorescence were monitored over time. As we can see in Figure S5, no degradation was observed for both.

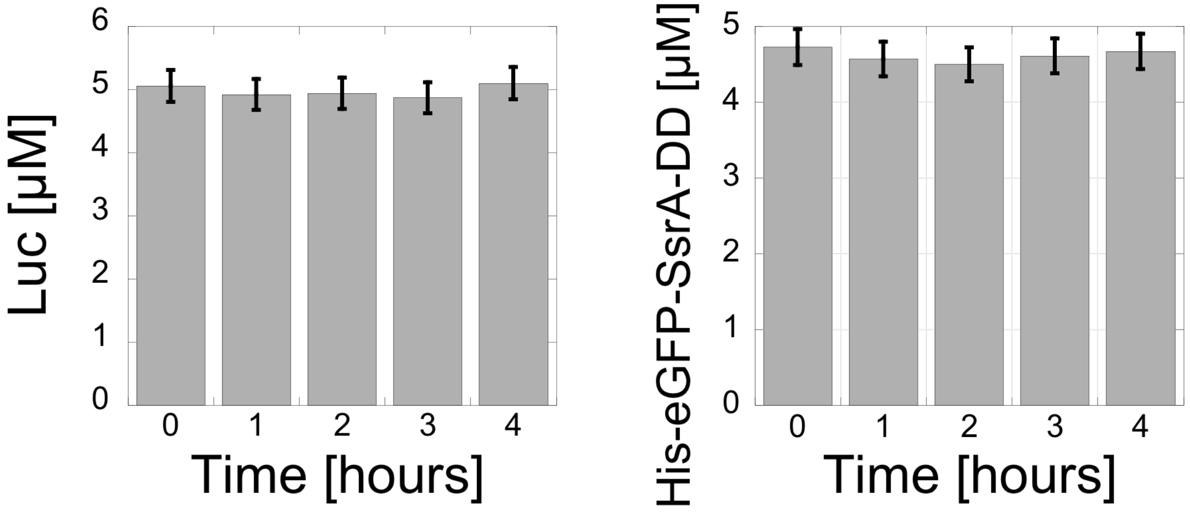

Figure S5

Stability of Luc and eGFP in a cell-free reaction. Recombinant pure Luc and pure His-eGFP-SsrA-DD were added into a cell-free reaction at a concentration of 5 µM. Luminescence and fluorescence were measured every hour.

Degradation of pure His-eGFP-SsrA in a cell-free reaction

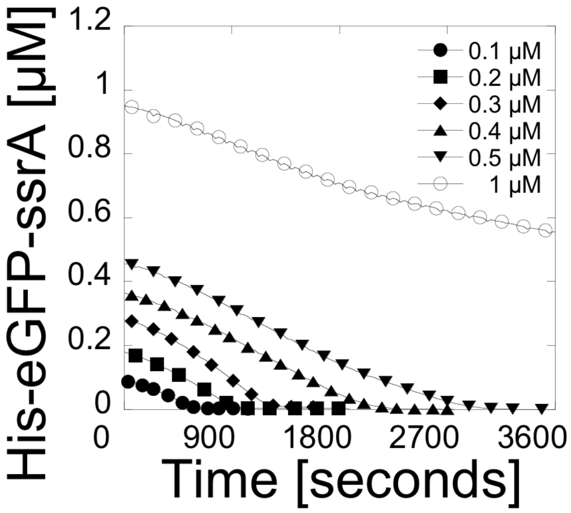

Figure S6

Degradation of His-sGFP-SsrA in a cell-free reaction. Kinetics of degradation of pure His-eGFP-SsrA protein at different concentrations in a cell-free reaction.

Protein degradation in a T7 cell-free system

A cell-free expression system with the T7 RNAP for transcription was used to test the degradation of eGFP tagged with 6 different AAA+ degrons.

Plasmids used:
pIVEX2.3d-eGFP
pIVEX2.3d-eGFP-SsrA/DD
pIVEX2.3d-eGFP-SsrA/D
pIVEX2.3d-eGFP-Crl
pIVEX2.3d-eGFP-YbaQ
pIVEX2.3d-eGFP-SsrA

The plasmid concentration was fixed to 2 nM. The experiment was carried out at 30°C, on plate reader. As we can see in the Figure S7, the results obtained with a T7 system are comparable to the results obtained with the extract used in the study (transcription with the *E. coli* RNA polymerase and sigma factor 70, see Figure 4A and 4B). No major differences were observed.

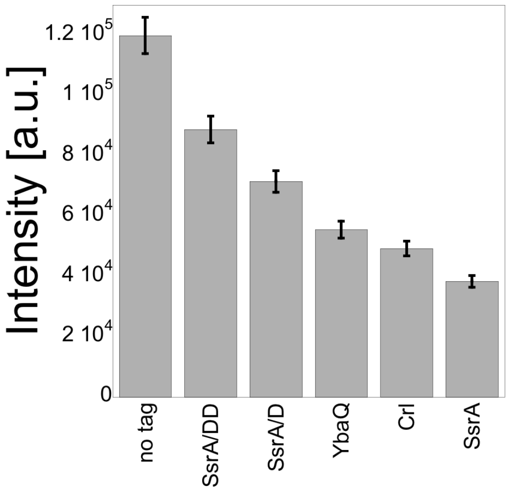

Figure S7
Cell-free expression as a function of the AAA+ degron used in an extract with the T7 bacteriophage RNA polymerase for transcription. End-point measurements of eGFP synthesized as a function of the tag used. Fluorescent intensity was measured on plate reader after 4 hours.
